# Near-InfraRed PhotoImmunoTherapy (NIR-PIT) for the local control of solid cancers: Challenges and potentials for human applications

**DOI:** 10.1016/j.critrevonc.2021.103325

**Published:** 2021-05

**Authors:** Irene Paraboschi, Stephen Turnock, Gabriela Kramer-Marek, Layla Musleh, Marta Barisa, John Anderson, Stefano Giuliani

**Affiliations:** a Wellcome/EPSRC Centre for Interventional & Surgical Sciences, University College London, London, UK; b Division of Radiotherapy and Imaging, The Institute of Cancer Research, London, UK; c Department of Specialist Neonatal and Pediatric Surgery, Great Ormond Street Hospital for Children NHS Foundation Trust, London, UK; d Cancer Section, Developmental Biology and Cancer Programme, UCL Great Ormond Street Institute of Child Health, London, UK; e Department of Oncology, Great Ormond Street Hospital for Children NHS Foundation Trust, London, England, UK

**Keywords:** PhotoImmunoTherapy, Near-InfraRed fluorophores, Monoclonal antibodies, Solid cancers, Local control, *In vivo*, *In vitro*, Clinical trials

## Abstract

Near-InfraRed PhotoImmunoTherapy (NIR-PIT) is a novel cancer-targeted treatment effected by a chemical conjugation between a photosensitiser (*e.g.* the NIR phthalocyanine dye IRDye700DX) and a cancer-targeting moiety (*e.g.* a monoclonal antibody, moAb). Delivery of a conjugate *in vivo* leads to accumulation at the tumour cell surface by binding to cell surface receptors or antigens. Upon deployment of focal NIR-light, irradiation of the conjugate results in a rapid, targeted cell death. However, the mechanisms of action to produce the cytotoxic effects have yet to be fully understood. Herein, we bring together the current knowledge of NIR-PIT from preclinical and clinical studies in a variety of cancers highlighting the key unanswered research questions. Furthermore, we discuss how to enhance the local control of solid cancers using this novel treatment regimen.

## Highlights

• Near-InfraRed PhotoImmunoTherapy (NIR-PIT) is a promising technique aiming to selectively kill cancer cells while leaving the host tissues and organs undamaged.
• It is effected by a chemical conjugation between a photosensitiser (*e.g.* the NIR phthalocyanine dye IRDye700DX) and a cancer-targeting moiety (*e.g.* a monoclonal antibody, moAb).
• Preclinical and clinical studies of NIR-PIT in a variety of cancers are here brought together highlighting the key unanswered research questions.

## Graphical abstract

**Figure:**
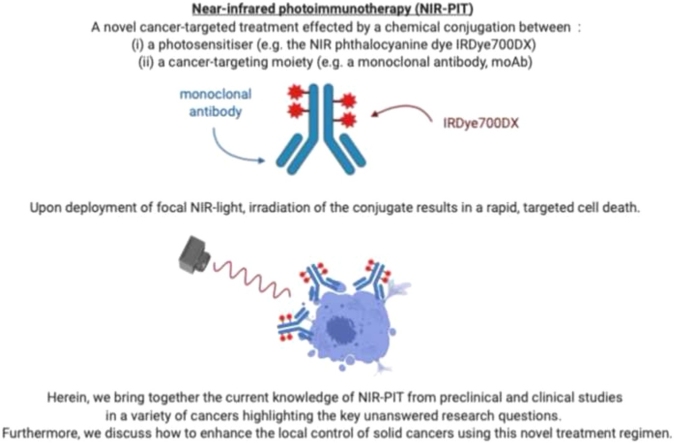

## 1 Introduction

For over half a century, cancer treatment has relied on the well-established triad of surgery, chemotherapy and radiotherapy. In particular, long-term oncological outcomes have been closely associated with the success or failure of complete surgical resection of the primary tumour (Nagaya et al., 2017a). Several studies have shown a clear correlation between subtotal microscopic resections and higher local recurrences in head and neck (Haque et al., 2019), urological (Wieder and Soloway, 1998; Dotan et al., 2007) and intestinal cancers (Nagtegaal and Quirke, 2008), culminating in poor patient outcomes. However, performing a complete tumour excision often presents a major challenge for surgeons. This is mainly due to the difficulty in clearing microscopic residual disease at the margins of resection, in particular when the tumours infiltrate or encase vital organs and vasculature. This limitation is common among all the surgical specialities and there is a need for novel adjuvant treatments capable of consolidating loco-regional control to reduce the risk of disease progression and metastatic spread.

External beam radiotherapy (EBR), chemotherapy and immunotherapy are common adjuvant treatments to eradicate residual cancer cells after surgery, but have significant limitations. Briefly, the disadvantages of EBR include damage to healthy surrounding tissues, failure to treat tumour areas that are not included in the radiation field, and increased incidence of secondary malignancy (De Ruysscher et al., 2019). Chemo- and immunotherapy are systemic consolidation approaches towards residual cancer cells, but the therapy can also be associated with significant side effects (*e.g.* cytokine release syndrome, inflammatory response, skin rash, colitis) and lead to autoimmune disorders (Su et al., 2020; Amos et al., 2011).

Photodynamic therapy (PDT) is a non-selective treatment method based on the combination of a photosensitive compound (photosensitiser, PS), visible light and tissue oxygen, none of which is toxic to cells or tissues by itself. The activation of a light-absorbing compound initiates processes leading to the destruction of the target cells (van Straten et al., 2017; Kwiatkowski et al., 2018). Nonetheless, delivery of the photosensitiser in PDT lacks tumour specificity and off-target and cutaneous toxicities may be observed.

Near-InfraRed PhotoImmunoTherapy (NIR-PIT) is emerging as a promising technique aiming to selectively kill cancer cells while leaving the host tissues and organs undamaged (Kobayashi and Choyke, 2019). The conjugation of the PS to an antigen specific monoclonal antibody (moAb) enables selective targeting the tumour, reducing potential side effects from NIR-PIT compared to PDT (Kobayashi et al., 2020a).

Here we present current evidence on NIR-PIT mechanisms of action and discuss the most recent preclinical and clinical studies demonstrating the utility of this novel adjuvant treatment.

## 2 Near-infrared photoimmunotherapy: mechanisms of action

In NIR-PIT the PS is conjugated to a highly specific monoclonal antibody (moAb) that has the ability to engage the selected target of interest, permitting an enhanced tumour specificity over PDT. Recent developments in antibody engineering technology have led to a new wave of PIT-suitable agents that are built on a range of moAbs, fragments and affibodies (Watanabe et al., 2015; Wei et al., 2020; Yamaguchi et al., 2019). Various PSs have been tested for NIR-PIT purposes and the silicon phthalocyanine dye, IRDye700DX has, so far, shown the most favourable properties (Kobayashi and Choyke, 2019). IRDye700DX has three diamagnetic silicons, which make it an efficient producer of singlet oxygen, and a high photostability compared to other commonly used dyes (Sato et al., 2018).

The highly selective cancer cell death induced by the NIR-PIT could be explained by the following non mutually exclusive mechanisms of action:

### 2.1 Oxidative stress

Conventional PDT has primarily worked through the production of singlet oxygen species (SOS) and reactive oxygen species (ROS) following NIR irradiation which are responsible for the necrotic cell death (van Straten et al., 2017; Kwiatkowski et al., 2018). However, the mechanisms by which SOS and ROS contribute to NIR-PIT induced cell death remain a matter of debate among experts in the field. In 2017, Railkar et al. (Railkar et al., 2017) showed that NIR-PIT produced significant levels of SOS and ROS in both *in vitro* and *in vivo* experiments performed by using UMUC-5 cells, a bladder squamous cell carcinoma (SCC) line. Moreover, they were able to completely rescue the NIR-PIT induced cell death with the addition of antioxidant agents such as NaN_3_ (SOS quencher) and Trolox (ROS quencher).

On the other hand, the treatment with SOS (NaN_3_) and ROS (*i.e.* N-acetyl cysteine, glutathione, 4-hydroxy TEMPO) scavengers only partially inhibited the phototoxicity effects of NIR-PIT in the experimental studies run by Mitsunaga et al. (Mitsunaga et al., 2011) and by Jin et al. (Jin et al., 2016) using 3T3/HER2 and MDA-MB-231 cells, respectively.

More recently, mass spectrometry (MS) was employed to investigate whether the oxidation of lipid molecules by ROS could be responsible for NIR-induced membrane disruption (Kobayashi and Choyke, 2019). Here, the analysis showed that the major component in the lipid membrane, the 16 − 1 phosphatidylcholine, was only minimally oxidised therefore suggesting possibly a minor role played by ROS in the physical stress of the cellular membrane. However, Kono et al. (2020) demonstrated IR700 modified liposomes were disrupted through oxidisation of cholesteral in the lipid membranes following NIR-irradiation. This was then inhibited through SOS (NaN_3_) scavenging. The authors also showed that liposome disruption was enhanced in deoxygenated conditions, suggesting alternative mechanisms are taking place instead of oxidative stress. Further studies are needed to elucidate the effect of SOS/ROS on the cell membrane disruption and the subsequent cell death.

### 2.2 Axial ligand-release

**Fig. 1:**
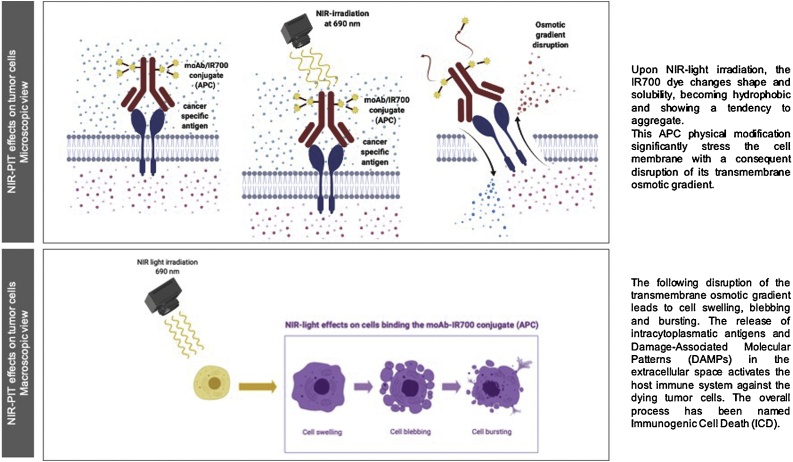
Direct cytotoxic effects of NIR-PIT on tumour cells expressing cancer specific antigens. **1A)** Upon NIR-light irradiation, the IR700 dye changes shape and solubility, becoming hydrophobic and showing a tendency to aggregate. This APC physical modification significantly stress the cell membrane with a consequent disruption of its transmembrane osmotic gradient. **1B)** The following disruption of the transmembrane osmotic gradient leads to cell swelling, blebbing and bursting. The release of intracytoplasmatic antigens and Damage-Associated Molecular Patterns (DAMPs) in the extracellular space activates the host immune system against the dying tumour cells. The overall process has been named Immunogenic Cell Death (ICD).

**Fig. 2:**
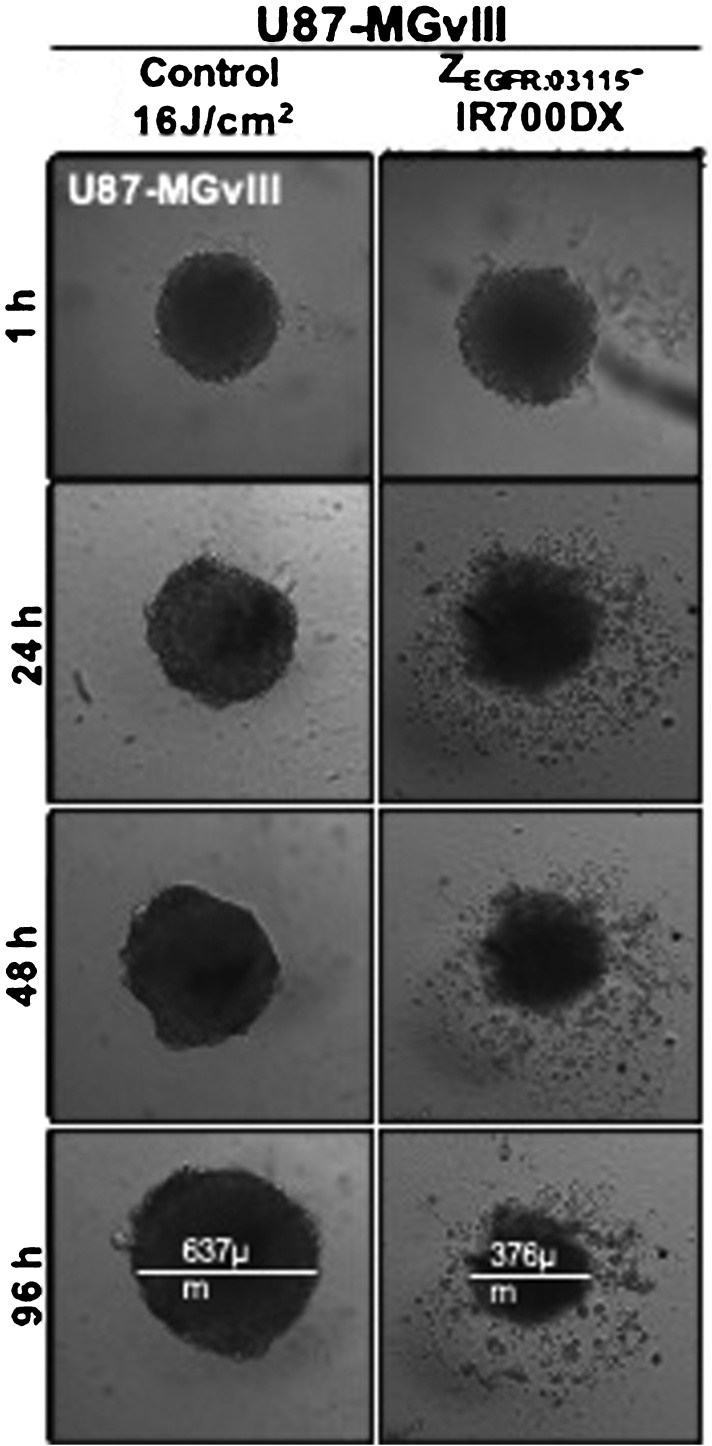
*In vitro* morphological changes following affibody-based PIT. Incubation of U87-MGvIII spheroids with the ZEGFR:03115–IR700DX for 6 h and irradiation with a red LED (16 J/cm2) induced phototoxic cell death and disintegration of the architectural structure of the spheroid population (Courtesy of Dr Gabriela Kramer-Marek) (For interpretation of the references to colour in this figure legend, the reader is referred to the web version of this article).

Sato et al. (Sato et al., 2018) have detailed physical changes produced by the moAb-IR700 conjugate when bound to the cancer cell surface. These modifications, initiated by NIR-light, may cause physical stress and disruption of the cell membrane. MS analyses have shown that an axial ligand (C_14_H_34_NO_10_S_3_Si) is released from the IR700 after irradiation at 690 nm. Using frequency modulation-atomic force microscopy (FM-AFM) and gel-electrophoresis analyses, the authors demonstrated changes in the hydrophilicity of the conjugate with consequent alteration of its shape and solubility. This led to a propensity of the moAb-IR700 conjugate to aggregate, leading to the disruption of the transmembrane osmotic gradient (Fig. 1). As a result, cells began swelling, blebbing and bursting (Fig. 2), as demonstrated by 3D dynamic low coherence quantitative phase microscopy (3D LC-QPM) and dual-view inverted selective plane illumination microscopy (diSPIM).

Recently, Kobayashi et al. (Kobayashi et al., 2020b) have provided a theoretical mechanism by which the IR700 axial-ligand is released. Through NIR irradiation, a radical anion form of IR700 is produced upon which water molecules cleave a central Si—O bond through an acid-base protonation reaction. This hydrolysis reaction releases the Si axial-ligand and results in cell damage. This mechanism is supported by previous work by Anderson et al. (2016), 2018 thereby the authors also demonstrated that activating a silicon phthalocyanine by 690 nm light in hypoxic conditions resulted in cell death in adjacent, non-irradiated target cells. Therefore, the axial ligand release may be the primary damaging mechanism in hypoxic tissue, whereby the production of ROS and SOS is compromised by the low tissue oxygen levels.

### 2.3 Host anti-cancer immune response

**Fig. 3:**
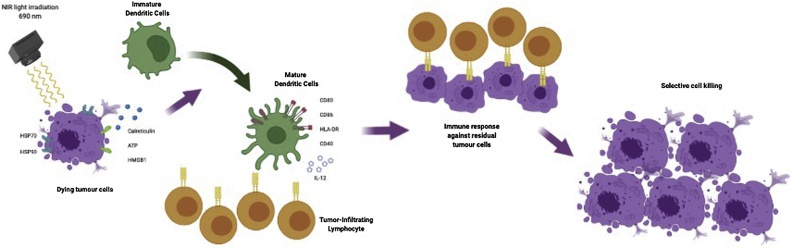
Cytotoxic effects of NIR-PIT induced by the activation of the host anti-cancer immune response. Following the exposure to NIR light, dying tumour cells rapidly release cancer-specific antigens and membrane damage danger signals which promote the maturation of Dendritic Cells. The consequent activation of tumour infiltrating CD8 + T cells result in the selective killing of residual tumour cells.

Irrespective of the mechanism responsible for the NIR-induced cell death, the consequent swelling and bursting represent an uncontrolled and rapid cell death. This phenomenon inherently alerts the immune system to danger in contrast with the controlled, non-immunogenic apoptotic pathway of cell death. Rapid cell lysis leads to the release of cytoplasmic antigens and damage-associated molecular patterns (DAMPs) in the extracellular space, which activates the host immune system against the dying tumour cells (Garg et al., 2010). Consequently, the direct cytotoxic effects of NIR-light irradiation are further enhanced by the activation of the host anti-cancer immune response (Kobayashi and Choyke, 2019; Ogawa et al., 2017; Nagaya et al., 2019). Dendritic cells (DCs) are fundamental cells in the adaptive and innate immune system. Garg et al. (2012) have shown that DCs increase the expression of maturation and costimulatory markers (*i.e.* CD80, CD86, CD40 and HLA-DR) when in co-culture with NIR-PIT treated tumour cells. Then mature DCs prime and educate naïve T cells leading to proliferation and cell-mediated cancer cell killing. DCs upregulate the transcription of the IL-12 cytokine gene, which promotes the development of a Th1 response and this is a potent inducer of IFNγ production by T cells and NK cells (Ogawa et al., 2017). As a consequence of DC maturation, a systemic polyclonal T-cell response was initiated by NIR-PIT during *in vivo* studies and resulted in complete tumour rejection at both primary and distant sites when combined with PD-1 checkpoint blockade (Nagaya et al., 2019) (Fig. 3).

## 3 NIR-PIT: preclinical studies

Several preclinical feasibility mouse studies have investigated potential breakthroughs of NIR-PIT for the treatment of solid tumours. The most significant are grouped and illustrated based on the body system involved, as reported in Table 1. The majority of studies have been performed using human tumour systems in immunodeficient mice where there can be no contribution of host adaptive immune system to tumour control. We therefore categorise studies as those performed syngeneic immune competent hosts or immunodeficient xenograft studies and identify the mouse backgrounds per study. In mice such as NSG lacking T, B and NK cells, tumour control efficacy can be attributable to direct killing of tumour cells but without the involvement of the immune system.

**Table 1:** The main preclinical feasibility studies investigating the potential breakthroughs of NIR-PIT for the treatment of solid tumors.

	Cancer type	Antigen targeted	Conjugated	Cell lines used in *in vivo* experiments	Mice models	Amount of conjugated injected	NIR light dose and timing
**Urological tumors**							
(Railkar et al., 2017)	Bladder cancer	EGFR	Anti-EGFR moAb (Panitumumab)	UMUC-5; UMUC-3	Athymic Nu/Nu mice	120 ug once (sc model)	100 J/cm^2^ (day 1) + 50 J/cm^2^ (day 2)
(Kiss et al., 2019)	Bladder cancer	CD47	B6H12	639 V	Immuno-compromised NSG (Nod.Cg-PrkdcscidIl2rgtm 1Wj1/SzJ) mice	200 ug once (sc model)	100 J/cm^2^ (day 1) + 50 J/cm^2^ (day 2);
						200 ug weekly for 5 weeks (sc model)	100 J/cm^2^ (day 1) + 50 J/cm^2^ (day 2) (at week 1), 100 J/cm^2^ (day 1) only (for week 2–5)
(Nagaya et al., 2017b)	Prostate cancer	PMSA	Anti-PMSA moAb	PC3	Athymic nude mice	100 μg weekly for 3 weeks (sc model)	50 J/cm^2^ (day 1) + 100 J/cm^2^ (day 2) (for 3 weeks)
**Gastrointestinal tumors**							
(Maawy et al., 2015)	Pancreatic cancer	CEA	Anti-CEA moAb	BxPC-3	Athymic nu/nu nude mice	100μ g (orthotopic model)	270 J/cm^2^ (day 1)
(Hanaoka et al., 2015)	Liver cancer	GPC3	YP7; HN3	A431/G1	Athymic nude mice	68.5 μ g (HN3) (sc model)	50 J/cm^2^ (day 1) + 100 J/cm^2^ (day 2) + 100 J/cm^2^ (day 3)
						100 μg (YP7) (sc model)	50 J/cm^2^ (day 1) + 100 J/cm^2^ (day 2) + 100 J/cm^2^ (day 3)
Sato et al., 2014	Gastric peritoneal carcinomatosis	HER2	Anti-HER2 moAb (Trastuzumab)	N87	Athymic nude mice	100 μg weekly for 3 weeks (sc model)	50 J/cm^2^ (day 1) + 100 J/cm^2^ (day 2) (for 3 weeks)
						100 μg (orthotopic model)	50 J/cm^2^ (day 1) + 100 J/cm^2^ (day 2)
(Wei et al., 2020)	Colorectal Tumors	GPA33	A33scFv antibody	LS174 T, COLO205	BALB/C nu/nu mice	100 μg	120 J/cm^2^ (hour 4)
**Lung tumors**							
(Sato et al., 2015a)	Non-Small Cell Lung Carcinoma	HER2	Anti-HER2 moAb (Trastuzumab)	Calu3	Athymic nude mice	100 μg (sc model)	50 J/cm^2^ (day 1) + 100 J/cm^2^ (day 2)
						100 μg (orthotopic model)	50 J/cm^2^ (day 1) + 100 J/cm^2^ (day 2)
(Nagaya et al., 2017c)	Papillary adenocarcinoma of the lung	PD-L1	Anti-PD-L1 moAb (Avelumab)	H441	Athymic nude mice	100 μg (sc model)	50 J/cm^2^ (day 1) + 100 J/cm^2^ (day 2)
(Nakamura et al., 2017)	Lung cancer expressing human EGFR	EGFR	Anti-EGFR moAb (Panitumumab)	Transgenic mouse model of spontaneous lung cancer expressing human EGFR	Double transgenic doxycycline inducible mice	150 ug (orthotopic model)	40 J/cm^2^ (day 1)
(Sato et al., 2015b)	Lung metastasis	HER2	Anti-HER2 moAb (Trastuzumab)	3T3/HER2	Athymic nude mice	100 μg (sc model)	100 J/cm^2^ (day 1)
						100 μg (orthotopic model, one shot regimen)	100 J/cm^2^ (day 1)
						100 μg every other day for 4 days (orthotopic model, four shot regiment)	100 J/cm^2^ (day 1) (following each moAb administration)
(Sato et al., 2015c)	Lung metastasis	HER2	Anti-HER2 moAb (Trastuzumab)	3T3/HER2	Athymic nude mice	100 μg (orthotopic model)	50 J/cm^2^ (day 1)
**Head and neck tumors**							
(Nagaya et al., 2017d)	Oral Cavity Squamous Cell Carcinoma (OSCC)	CD44	Anti-CD44	MOC1, MOC2-luc, MOC2-mKate2	C57BL/6 mice	100 μg (unilateral sc model)	50 J/cm^2^ (day 0) + 100 J/cm^2^ (day 1)
						100 μg (bilateral sc model)	100 J/cm^2^ (day 0)
**Gynecological tumors**							
(Sato et al., 2015d)	Disseminated peritoneal ovarian cancer	HER2	Anti-HER2 moAb (Trastuzumab)	SKOV	Athymic nude mice	100 μg (sc model)	100 J/cm^2^ (day 1)
						100 μg (orthotopic model)	100 J/cm^2^ (day 1)
(Nagaya et al., 2015)	Triple-negative breast cancer	EGFR	Anti-EGFR moAb (Cetuximab)	MDAMB231, MDAMB468	Athymic nude mice	300 μg (sc model, one shot regimen)	50 J/cm^2^ (day 1) + 100 J/cm^2^ (day 2)
						100 μg + 50 μ g (the following day) every week for 2 weeks (sc model, two split regimen)	50 J/cm^2^ (day 1) + 100 J/cm^2^ (day 2) (every week for 2 weeks)
						100 μg every week for 3 weeks (sc model, three split regimen)	50 J/cm^2^ (day 1) + 100 J/cm^2^ (day 2) (every week for 3 weeks)
(Jin et al., 2016)	Triple-negative breast cancer	CD44	Anti-CD44 moAb	MDA-MB-231, BT-474	Athymic Balb/c (nu/nu) mice	100 μg (sc model, one shot regimen)	30 J/cm^2^ (day 1)
						300 μg (sc model, one shot regimen)	30 J/cm^2^ (day 1)
						100 μg every week for 2 weeks (sc model, two shot model)	30 J/cm^2^ (day 1)
(Yamaguchi et al., 2019)	Breast cancer	HER2	HER2 Affibody	SK-BR3, BT474, MDA-MB361	na	na	na
**Brain tumors**							
(Jing et al., 2016)	Glioblastoma	AC133/CD133	Anti-AC133 moAb	CD133-OE U251, NCH421k	Immunocompromised nude mice	100 μg (sc model)	100 J/cm^2^ (day 1)
						100 μg (orthotopic model)	50 J/cm^2^ (day 1) + 100 J/cm^2^ (day 3)
(Burley et al., 2018)	Glioblastoma	EGFR	Anti-EGFR affibody	U87-MGvIII	NCr athymic mice	18 μg (sc model)	100 J/cm^2^ (hour 1)

Abbreviations. Sc: subcutaneous.

### 3.1 Urological tumours

Bladder cancer (BC) is the sixth most common tumour worldwide. Although the majority of patients present with early-stage disease, BC recurrence is one the highest among all solid tumours, likely due to the persistence of minimal residual disease after primary surgery (Burger et al., 2013; Sylvester et al., 2006). The epidermal growth factor receptor (EGFR) is overexpressed in up to 74 % of BC tissue specimens (Chaux et al., 2012). EGFR has relatively low expression in the normal urothelium (Røtterud et al., 2005), and its predominant luminal location in urothelial tumours (Messing, 1990) has therefore made the antigen a very promising therapeutic target for locally-advanced BCs. Railkar et al. (Railkar et al., 2017) first investigated the role of NIR-PIT as a new selective therapeutic strategy for EGFR-positive BCs in 2017. Mouse xenograft models of a subcutaneous EGFR-overexpressing BC xenograft (UMUC-5) showed a significant attenuation of tumour growth when treated with 120 μg of anti-EGFR-IR700 (panitumumab-IR700) and irradiated at 24 h (100 J/cm^2^) and 48 h (50 J/cm^2^) post-administration; with a third of tumours completely regressing. Further, the authors showed no effect on tumour growth in immunodeficient mice bearing EGFR-low expressing BC xenografts (UMUC-3) with the same treatment regimen.

The role of anti-CD47 NIR-PIT in human BC cell lines (UMUC-3; HT-1376; 639 V) were also reported by Kiss et al. (Kiss et al., 2019), where the authors further demonstrated NIR-PIT induced cell death in BC primary cell lines derived from fresh surgical samples from 5 patients undergoing bladder cancer surgery. CD47 is a surface protein widely expressed on BCs, but absent in terminally differentiated luminal umbrella cells (Chan et al., 2009). Using subcutaneous xenograft models in NSG mice of CD47-overexpressing BCs, Kiss et al. (Kiss et al., 2019) showed that a single treatment with 200 μg of anti-CD47-IR700 followed by 100 J/cm^2^ (on day 1) and 50 J/cm^2^ (on day 2) of NIR-light resulted in a reduction of tumour growth compared with control mice receiving no treatment, anti-CD47-IR700 only or NIR-light only. Implementing this regimen over a 5-week treatment period, by repeating the injection of 200 μg of anti-CD47-IR700 followed by 100 J/cm^2^ (on day 1), NIR-PIT treatment further slowed down tumour growth and improved mouse survival in comparison to untreated controls or mice receiving only the anti-CD47-IR700 conjugate. Since antibodies targeting CD47 are an emerging field of immune-oncology through the promotion of tumour phagocytosis, it will be of interest to determine the extent to which macrophages are involved in tumour clearance following PIT (Smolle and Pichler, 2017).

Prostate cancer (PC) is the most common malignancy in men and the third cause of cancer-related death in the United States (Siegel et al., 2017). Amongst all PC specific markers, anti–prostate-specific membrane antigen (PMSA) is one of the most studied targets due to its abundant expression in nearly all PCs, especially in the most poorly differentiated, more aggressive and hormone-refractory subtypes (Nagaya et al., 2017b). Anti-PSMA-IR700 PIT has been reported preclinically and shown to inhibit tumour growth in athymic nuce mice bearing xenograft PSMA-positive PC tumours. After receiving three cycles of 100 μg of anti-PSMA-IR700 followed by the exposure to 50 J/cm^2^ (on day 1) and 100 J/cm^2^ (on day 2) of NIR-light, there was also an associated prolonged survival compared to the control mice (Nagaya et al., 2017b). The promising results achieved by this study advocate more investigations to confirm the role of anti-PSMA NIR-PIT as a new treatment modality for PCs, especially for early-stage diseases and loco-regional pelvis recurrences.

### 3.2 Gastro-intestinal tumours

Due to their late presentation and their indolent course, gastro-intestinal malignancies are usually lethal and associated with a high rate of local recurrence and distant spreading (Maawy et al., 2015).

One of the most aggressive gastro-intestinal tumours is pancreatic cancer. In this regard, a significant decrease in tumour size was reported in athymic nude mice bearing carcinoembryonic antigen (CEA)-positive orthotopic pancreatic cancers treated with 100 μg of anti-CEA-IR700 and exposed to 270 J/cm^2^ NIR light (690 nm) 24 h later (Maawy et al., 2015).

The common multifocal nature of hepatocellular carcinoma (HCC) makes it one of the best candidates for targeted drugs and high expression of glypican-3 (GPC3) in HCC makes NIR-PIT a very appealing treatment strategy for this tumour. Hanaoka et al. (Hanaoka et al., 2015) compared the efficacy of a whole anti-GPC3 IgG antibody (YP7) to a genetically engineered small heavy-chain anti-GPC3 antibody (NH3) for NIR-PIT in athymic nude mouse models of HCC. Under the same experimental conditions, comparable cell death was seen for both antibodies *in vitro* over a wide range of energies and *in vivo* using a regime of 50, 100 and 100 J/cm^2^ NIR-PIT on three consecutive days. Interstingly, the smaller NH3-IR700 conjugate showed a more favourable pharmacokinetic profile with a more homogeneous distribution within the tumour, indicating higher penetration of HN3. Further, the authors showed a lower re-accumulation of NH3-IR700 compared to YP7-IR700 24 h after the first NIR-light exposure. All these findings indicated that NH3 could be a promising platform for designing molecularly targeted agents against HCC.

NIR-PIT has also shown some promising results for the treatment of the peritoneal carcinomatosis usually associated with advanced gastric cancers (Sato et al., 2014). Using GFP-transduced N87-GFP cells, Sato et al. (Sato et al., 2014) highlighted the efficacy of 100 μg of anti-HER2-IR700 conjugate (trastuzumab-IR700) in treating HER2 antigen-positive disseminated peritoneal gastric cancers in athymic nude mice. The authors showed a significant reduction in the tumour GFP fluorescence intensity when exposed to 50 J/cm^2^ (on day 1) and 100 J/cm^2^ (on day 2) after the initial drug injection.

Glycoprotein A33 antigen (GPA33) is highly expressed in over 95 % of human colorectal cancers, the second leading cause of cancer-related death worldwide (Bray et al., 2018). The efficacy of NIR-PIT in combination with a single-chain antibody variable fragments for GPA33 (A33scFv) has been investigated in a recent study published by Wei et al. (2020). The A33scFv displayed specific binding to GPA33-positive colorectal tumour cells (LS174 T) *in vitro* and a single round of NIR-PIT (100 μg of A33scFv-IR700 iv + 120 J/cm^2^ NIR-light 4 h post-injection) markedly suppressed tumour growth without apparent systematic off-targeted effects *in vivo* experiments employing BALB/C nu/nu mice. Their results suggested that scFv-IR700 conjugates may represent an attractive approach for tumour treatment, increasing tumour penetration and allowing rapid systemic clearance, in this way reducing the risks of side-effects.

### 3.3 Lung tumours

Pulmonary cancer is the most common cause of tumour-related death worldwide while the lung is the most common site of spreading of distant metastases (Sato et al., 2015a). About 80 % of lung cancers are histologically classified as non-small cell lung carcinoma (NSCLC), which patient prognosis is further worsened by the frequent occurrence of pleural metastasis in more advanced disease (Sato et al., 2015a).

NIR-PIT effects in models of pleural disseminated HER2-positive NSCLCs (Ca-lu3-luc-GFP cells) were investigated by Sato et al. (Sato et al., 2015a). By using the Olympus BF XP-60 fluorescent thoracoscopy, a significant decrease in the GFP-fluorescence and IR700-fluorescence intensity was observed as the result of the transcutaneous application of NIR-light (50 J/cm^2^ on day 1 and 100 J/cm^2^ on day 2) in athymic nude mice receiving of 100 μg of anti-HER2-IR700 (trastuzumab-IR700). Notably, despite a small pleural effusion present in one out of seven mice, histological analyses excluded any apparent damage to the surrounding normal lung.

Moreover, Sato et al. (Sato et al., 2015b) have also reported that a single dose of anti-HER2 NIR-PIT (100 J/cm^2^ of NIR-light 24 h after the injection of 100 μg of trastuzumab-IR700) using a HER2-positive (3T3/HER2-luc-GFP) mouse model of lung metastases was sufficient to cause a significant reduction in the volume of tumour metastasis *in vivo.* However, multiple NIR-PIT exposures (100 μg of trastuzumab-IR700 on day 13, 15, 17 and 19 followed by 100 J/cm^2^ NIR-light 24 h post each APC injection) were required to improve the overall mouse survival.

A similar athymic nude mouse model of lung metastasis (consisting of 3T3-RFP, 3T3/HER2-luc-GFP and mixtures of 3T3-RFP and 3T3/HER2-luc-GFP) was also adopted to confirm the selective action of NIR-PIT on HER2-positive tumour cells (Sato et al., 2015c). No damage to the adjacent normal lung tissue or to non-targeted tumour cells was reported after the thoracic exposure to NIR-light at 50 J/cm^2^ 24 h after the tail vein injection of 100 μg of anti-HER2-IR700 conjugate.

Promising results were also achieved by Nakamura et al. (Nakamura et al., 2017) who investigated the therapeutic effects of NIR-PIT in a transgenic mouse model of spontaneously occurring EGFR-positive lung adenocarcinomas arising in mice with intact immune systems. Three cycles of 150 μg of anti-EGFR–IR700 (panitumumab-IR700) followed by the irradiation with NIR-light from two directions (each 20 J/cm^2^, *via* the back and the front) significantly reduced tumour growth as measured by serial Magnetic Resonance Imaging (MRI).

Furthermore, conjugates targeting programmed cell death-ligand 1 (PD-L1-IR700) induced significant therapeutic responses in subcutaneous models of lung papillary adenocarcinomas overexpressing PD-L1 antigens in athymic nude mice (Nagaya et al., 2017c). Anti-PD-L1-IR700 showed high tumour accumulation and high tumour-to-background ratio. Tumour growth was significantly inhibited by NIR-PIT treatment in mice receiving 100 μg of anti-PD-L1-IR700 i.v. and 50 J/cm^2^ NIR light 24 h post injection and 100 J/cm^2^ 48 h post injection compared with the other control groups (no treatment; APC i.v. only; NIR light only). This resulted in a significantly prolonged survival folowing NIR-PIT.

### 3.4 Head and neck tumours

Oral cavity squamous cell carcinoma (OSCC) is the sixth leading cause of cancer-related mortality worldwide. Its poor prognosis is most likely due to the advanced stage of disease at diagnosis and the high rate of loco-regional recurrence and distant metastasis (Kademani, 2007). The majority of head and neck tumours expresses CD44, a cancer stem-like antigen, for which targeting moAbs are currently under investigation in phase 1 clinical trials (Rupp et al., 2007; Sauter et al., 2007; Tijink et al., 2006; Menke-van der Houven van Oordt et al., 2016).

The immunological effects of anti-CD44 moAbs have been the focus of NIR-PIT studies involving syngeneic immuno-competent models of murine oral cancer (MOC), whose genetic alterations mirror human OSCC (Nagaya et al., 2017d). A significant tumour growth reduction associated with mouse survival improvements were seen after the irradiation with 50 J/cm^2^ and 100 J/cm^2^ of NIR-light, 24 and 48 h following the injection of 100 μg of CD44-IR700 conjugate, respectively. More interestingly, owing to the use of immunocompetent syngeneic mouse models and the use of bilateral subcutaneous MOC tumours, the authours were able to show a significant reduction in the size of the non-NIR-treated contralateral tumour as evidence of the activation of the host immune response.

### 3.5 Gynecological tumours

Ovarian carcinoma is the leading cause of gynaecological cancer-related deaths throughout the world (Nath et al., 2019). The advanced stage disease at diagnosis, the difficulty of the surgical excision due to the strict adhesion of cancer implants into the peritoneum and the relatively rapid onset of chemo-resistance have contributed to a minimal improvement of the 5-year overall survival (OS) observed in the last few decades (Nath et al., 2019). Therefore, there is a desperate need for novel targeted cancer therapies that can selectively kill tumour cells while causing minimal damage to the adjacent healthy tissue.

In this regard, Sato et al. (2015d) examined the cytotoxic effects of NIR-PIT in 3D spheroids composed of HER2-positive ovarian cancer cells. Repeated exposure to 2 J/cm^2^ of NIR-light ultimately killed tumour cells previously incubated with anti-HER2-IR700 (trastuzumab-IR700). These results were also confirmed by *in vivo* studies, whereby a dramatic decrease in tumour volume and luciferase activity of human ovarian SKOV3-luc-D3 cells engrafted in athymic nude mice was achieved by exposing flank or disseminated peritoneal ovarian cancers to 100 J/cm^2^ of NIR-light 24 h after the administration of 100 μg of the APC.

Promising results have also been reported for NIR-PIT by preclinical studies focusing on triple-negative breast cancers (TNBCs), one of the most aggressive malignancies in women (Nagaya et al., 2015). EGFR overexpression in up to 70 % of TNBCs makes anti-EGFR-IR700 (cetuximab-IR700) an ideal candidate for NIR-PIT (Nagaya et al., 2015). In this regard, a significant tumour suppression associated with a prolonged survival was shown in athymic nude mice bearing EGFR-positive human TNBCs treated with cetuximab-IR700 and exposed to NIR-light (50 J/cm^2^ on day 1 and 100 J/cm^2^ on day 2). Interestingly, compared with one dose only of cetuximab-IR700 (300 μg of cetuximab-IR700 i.v. plus NIR light irradiation), a “two-split” approach (100 μg of cetuximab-IR700 i.v. with a top-up dose of 50 μg of cetuximab-IR700 i.v. immediately after NIR light irradiation on day 1) and “three split” (100 μg of cetuximab-IR700 i.v. once per week plus NIR light irradiation after each dosing) regimens led to improved therapeutic outcomes with no side effects.

Similarly, CD44-IR700 NIR-PIT caused significant cell death and dramatic reduction of tumour growth in TNBCs overexpressing the CD44 antigen, a cancer stem cell (CSC) marker frequently found in aggressive breast carcinomas, as described by Jin et *al* in their *in vitro* and *in vivo* experiments employing athymic Balb/c (nu/nu) mice (Jin et al., 2016). Interestingly, in human breast CSCs, the up-regulation of the oxidative response genes in free radical scavenging systems leads to their resistance to apoptotic death from ROS-dependent therapies, such as PDT (Diehn et al., 2009). By testing the effects of ROS scavengers (N-acetyl cysteine; glutathione; 4-hydroxy TEMPO), only the broad-spectrum antioxidant N-acetyl cysteine partially inhibited CD44-IR700-induced cell death by approximately 20 %, implying that molecular oxygen did not play a major role in photocytotoxicity from CD44-IR700-mediated PIT.

Not only moAbs, but also small protein mimetic affibodies have been conjugated to the phthalocyanine dye IRDye700DX to induce the selective destruction of breast cancer cells when irradiated with NIR-light at 690 nm (Yamaguchi et al., 2019). The effects of NIR-PIT were well correlated with the level of HER2 protein expression on targeted cells. Furthermore, cell viability decreased in a NIR-light-dose and HER2 affibody-IR700 conjugate-concentration dependent manner. The employment of affibody molecules may offer unique opportunities in the clinical translation of NIR-PIT thanks to their rapid clearance and good tissue penetration. Moreover, Mączyńska et al. (2020) demonstrated that the administration of a IR700-based HER2-targeted affibody conjugate can drive immunogenic cancer cell death and influence the innate and adaptive anti-tumour immune response when irradiated with NIR-light. These findings suggest that affibody-based PIT is an attractive alternative to moAb-based options, particularly for patients whose tumours acquire resistance to conventional anti-HER2 therapies.

### 3.6 Brain tumours

Glioblastoma multiforme (GBM) is the most common primary brain tumour in adults. It shows particularly aggressive behaviour and invasiveness most likely due to the presence of CSCs in its advance front. AC133, an epitope of CD133, is a CSC marker for many malignancies, including GBM (Chen et al., 2010; Singh et al., 2004).

Interestingly, preclinical studies investigating the role of anti-AC133 NIR-PIT showed not only a significant reduction in tumour growth but also a delayed CSC-driven tumour initiation when subcutaneous CD133-positive GMB xenografts were treated with NIR-light (Jing et al., 2016). In fact, when 5 × 10^6^ cells pre-incubated for 14 h with 40 μg/mL of AC133-IR700 were bilaterally injected into the flanks of nude mice and unilaterally exposed to 100 J/cm^2^, tumour growth inhibition only occurred in the side exposed to NIR-light. Moreover, a dramatic decrease in tumour volume and a significant increase in survival were reported in mice bearing orthotopic CD133-positive GBM xenografts trans-cranially irradiated with 50 J/cm^2^ (on day 1) and 100 J/cm^2^ (on day 3) of NIR-light after the administration of 100 μg of AC133-IR700.

Further, EGFR amplification occurs in about 50 % of *de novo* primary GBMs, representing the most common genetic aberration in these patients. Recently, Burley et al. (2018) demonstrated that the administration of an affibody-NIR-activated conjugate targeting EGFR (Z_EGFR:03115_-IR700) selectively induces GBM cell death with no detected toxicity in normal tissues in orthotopic brain tumour models employing NCr athymic mice. The high binding affinity of this affibody molecule, its small size and excellent tumour penetration make the conjugate ideal targeting agents for GBM therapy. In particular, their ability to cross the blood-brain barrier can be beneficial to clearly image and efficiently treat not only the brain primary tumours but also secondary metastases.

## 4 NIR-PIT: clinical trials in humans

Based on the promising results of many preclinical studies, the first-in-human phase 1/2a clinical trial for NIR-PIT started in June 2015 in patients with intractable recurrent head and neck squamous cell carcinoma (HNSCC) involving seven cancer centers in the US (https://clinicaltrials.gov/ct2/show/NCT02422979). The first part of this trial focused on the establishment of a recommended dose of the experimental drug. A parenteral formulation of cetuximab-IR700 (RM-1929) was administrated at three different doses (160 mg/m^2^, 320 mg/m^2^, 640 mg/m^2^) 24 h prior to NIR-light irradiation (50 J/cm^2^ for superficial lesions or 100 J/cm^2^ for interstitial lesions).

The definition of the Drug Maximum Tolerated Dose (MTD), the Maximum Feasible Dose (MFD), the adverse event profile for each drug dose and the photo-safety of the applied NIR-light were the primary outcomes of this study, while the drug pharmacokinetic profile, the tumour response/reduction/necrosis and the immunogenic response were the secondary outcomes.

The second part of this trial investigated the safety and the anti-cancer efficacy of up to four repeated treatments of RM-1929 administered at the MFD (640 mg/m^2^), and activated with a fixed amount of NIR-light: (i) 75 J/cm^2^ for superficial lesions or 150 J/cm^2^ for interstitial lesions; (ii) 100 J/cm^2^ for superficial lesions or 200 J/cm^2^ for interstitial lesions; (iii) 50 J/cm^2^ for superficial lesions or 100 J/cm^2^ for interstitial lesions.

Preliminary results of this two-part study confirmed human safety and the efficacy of RM-1929 NIR-PIT. Both tumour response and patient survival (overall response rate of 44.8 %, median PFS of 5.7 months and median OS of 9.5 months) improved over conventional therapies in this highly selected group of patients (Gillenwater, ASCO 2018).

After receiving the Fast Track designation from the US Food and Drug Administration (FDA), a phase 3 randomised, double-arm clinical trial evaluating the effects of a parenteral formulation of cetuximab-IR700 (ASP-1929) started in May 2019 in 275 patients affected by recurrent HNSCC who have failed at least two lines of therapy (https://clinicaltrials.gov/ct2/show/NCT03769506). The trial is currently underway in the US, Belgium, Greece, Spain, Japan and Taiwan.

ASP-1929 efficacy will be disclosed by comparing the progression-free survival (PFS) and the OS of patients treated with repeated ASP-1929 PIT interventions (experimental arm) with those treated accordingly to the physician's choice standard of care (SOC), consisting of docetaxel, cetuximab, methotrexate or paclitaxel (control arm).

Moreover, in order to further improve its performance, NIR-PIT can be combined with conventional cancer immunotherapies, such as immune-checkpoint inhibitors. In this regard, a Phase 1b/2 clinical trial investigating the role of ASP-1929 administered in combination with anti-PD-L1 therapies in PD-L1 and EGFR-positive recurrent or metastatic HNSCCs (cohort 1) or PD-L1 and EGFR-positive locally advanced or metastatic cutaneous SCCs (cohort 2) is due to start in September 2020 (https://clinicaltrials.gov/ct2/show/NCT04305795). Anti-PD-L1 immunotherapy (200 mg of pembrolizumab in the cohort 1 and 350 mg of cemiplimab in the cohort 2) will be administered in addition to ASP-1929 to assess the safety, tolerability and tumour response of this combined regimen.

Alongside these APC studies, non-antibody mediated NIR-PIT has been employed in clinical trials investigating melanoma treatment. AU-011 is a novel established formulation of nanoparticles derived from the Human PapillomaVirus (HPV-NPs) conjugated to IR700. It selectively binds to specifically modified Heparan Sulphate ProteoGlycans (HSPGs) which are upregulated in ocular melanoma cells. An ascending single and repeat dose clinical trial investigating the safety, the immunogenicity and the efficacy of intravitreally injected AU-011 for the treatment of 57 patients with Small Primary Choroidal Melanoma started in February 2017 and is currently underway in the US (https://clinicaltrials.gov/ct2/show/study/NCT03052127). Preliminary safety and efficacy results in terms of treatment-related adverse events, local tumour control and visual acuity preservation seem to be very promising.

## 5 NIR-PIT: therapeutic benefits, limits and challenges and future perspectives

NIR-PIT offers several advantages over conventional cancer treatments.

Firstly, moAb NIR conjugates can be used as a specific tool to track tumour cells and response to treatment from diagnosis to follow-up. As well, the same conjugate can be used as intraoperative imaging to help the surgeon to achieve a safer and more radical resection (Kobayashi et al., 2020c). In fact, optical qualities of IRDye700DX fluorophore (such as the limited photobleaching, the water solubility and salt tolerance) make it an excellent optical probe for moAb conjugation. Due to the diagnostic and therapeutic properties of this dye, the moAb-based IRDye700DX conjugates can be considered as theranostic agents. In addition, fibro-optical diffusers could be inserted through endoscopes, trocars, needles and catheters in order to widely visualise and administer NIR-PIT to airways, gastro-intestinal or urinary tumours (Kobayashi and Choyke, 2019).

Secondly, APC targeted action provides a tumour-cell specificity with virtually no damage to normal adjacent structures given the appropriate choice of moAb specificity. These findings have been confirmed with both *in vitro* and *in vivo* studies, whereby free APC did not exert any detrimental effects on unbounded cells following NIR-light exposure. In fact, cell death efficiency was not increased by failing to wash cells from unbound conjugates prior to irradiation (Mitsunaga et al., 2011). By sparing adjacent normal cells, *e.g.* tumour-infiltrating immune cells, the innate and adaptive antitumour response can be further activated producing both local and systemic immune-activation. In this way, the tumour cell killing induced by the direct cytotoxicity effects of NIR-light irradiation is enhanced by the host anti-cancer immune response to DAMPs. The systemic antitumour immunity induced by NIR-PIT aids in the clearance of distant metastases and the prevention of tumour recurrences (Kobayashi and Choyke, 2019; Nagaya et al., 2019; Kobayashi et al., 2020c).

In addition, NIR-PIT exploits a form of non-ionising radiation. For this reason, no limits to its total cumulative dose have been reported, and multiple cycles of NIR-PIT could be safely employed.

Interestingly, increased permeability of tumour vessels follows the rapid NIR-PIT induced death of the perivascular cancer cells. This phenomenon, named super-enhanced permeability and retention (SUPR), could be employed to allow for more efficient delivery of chemotherapeutic agents into the tumour bed (Kobayashi and Choyke, 2019; Sano et al., 2013; Hanaoka et al., 2015). In fact, *in vivo* studies have demonstrated that NIR-PIT dramatically increased the therapeutic effects of liposome-containing daunorubicin (Sano et al., 2013) and nanosized albumin-bound-paclitaxel when compared to either therapy alone.

Finally, this new generation of APC drugs have a similar pharmacokinetic profile of the native naked moAbs, resulting in highly targeted tumour accumulation with minimal non-specific binding (Sato et al., 2015d). The unbound IR700 water-solubility following its dissociation from the targeted cells results in a rapid urinary excretion with no long-lasting photosensitising effects, as proved by the preliminary results of early clinical trials (Kobayashi and Choyke, 2019).

However, some limitations of NIR-PIT have yet to be settled. Firstly, NIR-light penetration up to 10 mm may limit the application of NIR-PIT in the case of large tumour masses, reducing therapeutic opportunities to the intraoperative clearance of minimal residual disease or lymph node metastases.

Secondly, a single tumour-specific antigen is not always overexpressed in spontaneously occurring cancers. However, this limitation could be overcome by the administration of a cocktail of APCs against different tumour antigens, resulting in a more homogeneous intratumoural micro-distribution (Nakajima et al., 2013). Finally, most studies have been carried out in immunodeficienet mice, most commonly athymic nude mice, and the contribution of host immune cells to tumour clearance has, therefore, not been appropriately investigated in most studies. The role of systemic antitumour immunity induced by NIR-PIT for the clearance of distant metastases and the prevention of tumour recurrences requires substantial further examination.

## 6 Conclusion

NIR-PIT is a recently developed molecularly-targeted cancer treatment that exploits a photochemistry-based process to kill tumour cells while selectively enhancing the host immune response. Preclinical feasibility studies and early results emerging from clinical trials have demonstrated the therapeutic potential of NIR-PIT, indicating this novel tool could become part of the armoury to improve loco-regional control and reduce the risk of microscopic residual disease with an overall benefit on survival.

## Authorship

Paraboschi Irene and Giuliani Stefano provided substantial contributions to the conception and design of the review. They also acquired, analysed and interpretationed data for the project.

Turnock Stephen and Kramer-Marek Gabriela revised the work critically for important intellectual content.

Musleh Layla and Barisa Marta agreed for all aspects of the work in ensuring that questions related to the accuracy or integrity of any part of the work were appropriately investigated and resolved.

Anderson John granted the final approval of the version to be published and revised the review critically.

## Funding

This work was supported by the Medical Research Council UK Clinical Academic Research Partnership (PI Stefano Giuliani, grant number MR/T005491/1) and by the Wellcome/EPSRC Centre for Interventional and Surgical Sciences (WEISS, grant number 203145Z/16/Z) at the University College London, United Kingdom.

## Declaration of Competing Interest

Nothing to declare.

## References

[bib0005] Amos S.M., Duong C.P.M., Westwood J.A. (2011). Autoimmunity associated with immunotherapy of cancer. Blood.

[bib0010] Anderson E.D., Gorka A.P., Schnermann M.J. (2016). Near-infrared uncaging or photosensitizing dictated by oxygen tension. Nat. Commun..

[bib0015] Anderson E.D., Sova S., Ivanic J., Kelly L., Schnermann M.J. (2018). Defining the conditional basis of silicon phthalocyanine near-IR ligand exchange. Phys. Chem. Chem. Phys. PCCP.

[bib0020] Bray F., Ferlay J., Soerjomataram I., Siegel R.L., Torre L.A., Jemal A. (2018). Global cancer statistics 2018: GLOBOCAN estimates of incidence and mortality worldwide for 36 cancers in 185 countries. CA Cancer J. Clin..

[bib0025] Burger M., Catto J.W.F., Dalbagni G. (2013). Epidemiology and risk factors of urothelial bladder cancer. Eur. Urol..

[bib0030] Burley T.A., Mączyńska J., Shah A. (2018). Near-infrared photoimmunotherapy targeting EGFR-Shedding new light on glioblastoma treatment. Int. J. Cancer.

[bib0035] Chan K.S., Espinosa I., Chao M. (2009). Identification, molecular characterization, clinical prognosis, and therapeutic targeting of human bladder tumor-initiating cells. Proc. Natl. Acad. Sci. U. S. A..

[bib0040] Chaux A., Cohen J.S., Schultz L. (2012). High epidermal growth factor receptor immunohistochemical expression in urothelial carcinoma of the bladder is not associated with EGFR mutations in exons 19 and 21: a study using formalin-fixed, paraffin-embedded archival tissues. Hum. Pathol..

[bib0045] Chen R., Nishimura M.C., Bumbaca S.M. (2010). A hierarchy of self-renewing tumor-initiating cell types in glioblastoma. Cancer Cell.

[bib0050] De Ruysscher D., Niedermann G., Burnet N.G., Siva S., Lee A.W.M., Hegi-Johnson F. (2019). Radiotherapy toxicity. Nat Rev Dis Primer.

[bib0055] Diehn M., Cho R.W., Lobo N.A. (2009). Association of reactive oxygen species levels and radioresistance in cancer stem cells. Nature.

[bib0060] Dotan Z.A., Kavanagh K., Yossepowitch O. (2007). Positive surgical margins in Soft tissue following radical cystectomy for bladder Cancer and Cancer Specific survival. J. Urol..

[bib0065] Garg A.D., Nowis D., Golab J., Vandenabeele P., Krysko D.V., Agostinis P. (2010). Immunogenic cell death, DAMPs and anticancer therapeutics: an emerging amalgamation. Biochim Biophys Acta BBA - Rev Cancer.

[bib0070] Garg A.D., Krysko D.V., Verfaillie T. (2012). A novel pathway combining calreticulin exposure and ATP secretion in immunogenic cancer cell death. EMBO J..

[bib0075] Hanaoka H., Nagaya T., Sato K. (2015). Glypican-3 targeted human heavy chain antibody as a drug carrier for hepatocellular carcinoma therapy. Mol. Pharm..

[bib0080] Haque S., Karivedu V., Riaz M.K. (2019). High-risk pathological features at the time of salvage surgery predict poor survival after definitive therapy in patients with head and neck squamous cell carcinoma. Oral Oncol..

[bib0085] Jin J., Krishnamachary B., Mironchik Y., Kobayashi H., Bhujwalla Z.M. (2016). Phototheranostics of CD44-positive cell populations in triple negative breast cancer. Sci. Rep..

[bib0090] Jing H., Weidensteiner C., Reichardt W. (2016). Imaging and Selective Elimination of Glioblastoma Stem Cells with Theranostic Near-Infrared-Labeled CD133-Specific Antibodies. Theranostics.

[bib0095] Kademani D. (2007). Oral cancer. Mayo Clin. Proc..

[bib0100] Kiss B., van den Berg N.S., Ertsey R. (2019). CD47-Targeted Near-Infrared Photoimmunotherapy for Human Bladder Cancer. Clin. Cancer Res. Off. J. Am. Assoc. Cancer Res.

[bib0105] Kobayashi H., Choyke P.L. (2019). Near-Infrared Photoimmunotherapy of Cancer. Acc. Chem. Res..

[bib0110] Kobayashi H., Griffiths G.L., Choyke P.L. (2020). Near-infrared photoimmunotherapy: photoactivatable antibody-drug conjugates (ADCs). Bioconjug. Chem..

[bib0115] Kobayashi M., Harada M., Takakura H. (2020). Theoretical and experimental studies on the near-infrared photoreaction mechanism of a silicon phthalocyanine photoimmunotherapy dye: photoinduced hydrolysis by radical anion generation. ChemPlusChem.

[bib0120] Kobayashi H., Furusawa A., Rosenberg A., Choyke P.L. (2020). Near-infrared photoimmunotherapy of cancer: a new approach that kills cancer cells and enhances anti-cancer host immunity. Int. Immunol..

[bib0125] Kono Y., Yokoyama K., Suzuki M., Takakura H., Ogawa M. (2020). Surface modification of liposomes using IR700 enables efficient controlled contents release triggered by Near-IR light. Biol. Pharm. Bull..

[bib0130] Kwiatkowski S., Knap B., Przystupski D. (2018). Photodynamic therapy - mechanisms, photosensitizers and combinations. Biomed. Pharmacother Biomed. Pharmacother.

[bib0135] Maawy A.A., Hiroshima Y., Zhang Y. (2015). Near infra-red photoimmunotherapy with anti-CEA-IR700 results in extensive tumor lysis and a significant decrease in tumor burden in orthotopic mouse models of pancreatic cancer. PLoS One.

[bib0140] Mączyńska J., Da Pieve C., Burley T.A. (2020). Immunomodulatory activity of IR700-labelled affibody targeting HER2. Cell Death Dis..

[bib0145] Menke-van der Houven van Oordt C.W., Gomez-Roca C., van Herpen C. (2016). First-in-human phase I clinical trial of RG7356, an anti-CD44 humanized antibody, in patients with advanced, CD44-expressing solid tumors. Oncotarget.

[bib0150] Messing E.M. (1990). Clinical implications of the expression of epidermal growth factor receptors in human transitional cell carcinoma. Cancer Res..

[bib0155] Mitsunaga M., Ogawa M., Kosaka N., Rosenblum L.T., Choyke P.L., Kobayashi H. (2011). Cancer cell-selective in vivo near infrared photoimmunotherapy targeting specific membrane molecules. Nat. Med..

[bib0160] Nagaya T., Sato K., Harada T., Nakamura Y., Choyke P.L., Kobayashi H. (2015). Near Infrared Photoimmunotherapy Targeting EGFR Positive Triple Negative Breast Cancer: Optimizing the Conjugate-Light Regimen. PLoS One.

[bib0165] Nagaya T., Nakamura Y.A., Choyke P.L., Kobayashi H. (2017). Fluorescence-guided surgery. Front. Oncol..

[bib0170] Nagaya T., Nakamura Y., Okuyama S. (2017). Near-Infrared Photoimmunotherapy Targeting Prostate Cancer with Prostate-Specific Membrane Antigen (PSMA) Antibody. Mol Cancer Res MCR.

[bib0175] Nagaya T., Nakamura Y., Sato K. (2017). Near infrared photoimmunotherapy with avelumab, an anti-programmed death-ligand 1 (PD-L1) antibody. Oncotarget.

[bib0180] Nagaya T., Nakamura Y., Okuyama S. (2017). Syngeneic mouse models of oral Cancer Are effectively targeted by Anti-CD44-Based NIR-PIT. Mol. Cancer Res. MCR.

[bib0185] Nagaya T., Friedman J., Maruoka Y. (2019). Host immunity following near-infrared photoimmunotherapy is enhanced with PD-1 checkpoint blockade to eradicate established antigenic tumors. Cancer Immunol. Res..

[bib0190] Nagtegaal I.D., Quirke P. (2008). What is the role for the circumferential margin in the modern treatment of rectal cancer?. J. Clin. Oncol. Off. J. Am. Soc. Clin. Oncol.

[bib0195] Nakajima T., Sano K., Choyke P.L., Kobayashi H. (2013). Improving the efficacy of Photoimmunotherapy (PIT) using a cocktail of antibody conjugates in a multiple antigen tumor model. Theranostics.

[bib0200] Nakamura Y., Ohler Z.W., Householder D. (2017). Near infrared photoimmunotherapy in a transgenic mouse model of spontaneous epidermal growth factor receptor (egfr)-expressing lung cancer. Mol. Cancer Ther..

[bib0205] Nath S., Saad M.A., Pigula M., Swain J.W.R., Hasan T. (2019). Photoimmunotherapy of ovarian cancer: a unique niche in the management of advanced disease. Cancers.

[bib0210] Ogawa M., Tomita Y., Nakamura Y. (2017). Immunogenic cancer cell death selectively induced by near infrared photoimmunotherapy initiates host tumor immunity. Oncotarget.

[bib0215] Railkar R., Krane L.S., Li Q.Q. (2017). Epidermal Growth Factor Receptor (EGFR)-targeted Photoimmunotherapy (PIT) for the Treatment of EGFR-expressing Bladder Cancer. Mol. Cancer Ther..

[bib0220] Røtterud R., Nesland J.M., Berner A., Fosså S.D. (2005). Expression of the epidermal growth factor receptor family in normal and malignant urothelium. BJU Int..

[bib0225] Rupp U., Schoendorf-Holland E., Eichbaum M. (2007). Safety and pharmacokinetics of bivatuzumab mertansine in patients with CD44v6-positive metastatic breast cancer: final results of a phase I study. Anticancer Drugs.

[bib0230] Sano K., Nakajima T., Choyke P.L., Kobayashi H. (2013). Markedly enhanced permeability and retention effects induced by photo-immunotherapy of tumors. ACS Nano.

[bib0235] Sato K., Choyke P.L., Kobayashi H. (2014). Photoimmunotherapy of gastric cancer peritoneal carcinomatosis in a mouse model. PLoS One.

[bib0240] Sato K., Nagaya T., Choyke P.L., Kobayashi H. (2015). Near infrared photoimmunotherapy in the treatment of pleural disseminated NSCLC: preclinical experience. Theranostics.

[bib0245] Sato K., Nagaya T., Mitsunaga M., Choyke P.L., Kobayashi H. (2015). Near infrared photoimmunotherapy for lung metastases. Cancer Lett..

[bib0250] Sato K., Nagaya T., Nakamura Y., Harada T., Choyke P.L., Kobayashi H. (2015). Near infrared photoimmunotherapy prevents lung cancer metastases in a murine model. Oncotarget.

[bib0255] Sato K., Hanaoka H., Watanabe R., Nakajima T., Choyke P.L., Kobayashi H. (2015). Near infrared photoimmunotherapy in the treatment of disseminated peritoneal ovarian cancer. Mol. Cancer Ther..

[bib0260] Sato K., Ando K., Okuyama S. (2018). Photoinduced ligand release from a silicon phthalocyanine dye conjugated with monoclonal antibodies: a mechanism of cancer cell cytotoxicity after near-infrared photoimmunotherapy. ACS Cent. Sci..

[bib0265] Sauter A., Kloft C., Gronau S. (2007). Pharmacokinetics, immunogenicity and safety of bivatuzumab mertansine, a novel CD44v6-targeting immunoconjugate, in patients with squamous cell carcinoma of the head and neck. Int. J. Oncol..

[bib0270] Siegel R.L., Miller K.D., Jemal A. (2017). Cancer Statistics, 2017. CA Cancer J. Clin..

[bib0275] Singh S.K., Hawkins C., Clarke I.D. (2004). Identification of human brain tumour initiating cells. Nature.

[bib0280] Smolle M.A., Pichler M. (2017). Inflammation, phagocytosis and cancer: another step in the CD47 act. J. Thorac. Dis..

[bib0285] Su C., Wang H., Liu Y. (2020). Adverse effects of Anti-PD-1/PD-L1 therapy in non-small cell lung Cancer. Front. Oncol..

[bib0290] Sylvester R.J., van der Meijden A.P.M., Oosterlinck W. (2006). Predicting recurrence and progression in individual patients with stage Ta T1 bladder cancer using EORTC risk tables: a combined analysis of 2596 patients from seven EORTC trials. Eur. Urol..

[bib0295] Tijink B.M., Buter J., de Bree R. (2006). A phase I dose escalation study with anti-CD44v6 bivatuzumab mertansine in patients with incurable squamous cell carcinoma of the head and neck or esophagus. Clin. Cancer Res. Off. J. Am. Assoc. Cancer Res.

[bib0300] van Straten D., Mashayekhi V., de Bruijn H.S., Oliveira S., Robinson D.J. (2017). Oncologic photodynamic therapy: basic principles, current clinical status and future directions. Cancers.

[bib0305] Watanabe R., Hanaoka H., Sato K. (2015). Photoimmunotherapy targeting prostate-specific membrane antigen: are antibody fragments as effective as antibodies?. J. Nucl. Med. Off. Publ. Soc. Nucl. Med..

[bib0310] Wei D., Tao Z., Shi Q. (2020). Selective Photokilling of Colorectal Tumors by Near-Infrared Photoimmunotherapy with a GPA33-Targeted Single-Chain Antibody Variable Fragment Conjugate. Mol. Pharm..

[bib0315] Wieder J.A., Soloway M.S. (1998). Incidence, etiology, location, prevention and treatment of positive surgical margins after radical prostatectomy for prostate cancer. J. Urol..

[bib0320] Yamaguchi H., Pantarat N., Suzuki T., Evdokiou A. (2019). Near-infrared photoimmunotherapy using a small protein mimetic for HER2-overexpressing breast cancer. Int. J. Mol. Sci..

